# Wounds of the Heart

**Published:** 1897-12-25

**Authors:** 


### Wounds of the Heart.

The murder of Mr. Terriss has directed public attention very strongly to the subject of wounds of the heart, and even in the midst of our regret at the unhappy event, professional interest is aroused by the important fact that, notwithstanding the extent of the wound, "which pierced the heart right through," the murdered man lived close upon an hour. The cases in which patients suffering from small wounds of the heart have lived for some time, and have even recovered, are by no means rare. A case was reported at the Clinical Society last year in which a man who had been stabbed over the third left costal cartilage, and had suffered severely from hæmorrhage, died 79 days after the injury from general causes, and after death a scar was found in the right ventricle showing that the heart had been penetrated.

But much more severe injuries of the heart may be recovered from. Muhlig relates the case of a man who was stabbed with a stiletto on the left side of the sternum. For a time his life was despaired of, but he recovered, and returned to his employment; and on his death, from other causes, ten years later, it was found that the pericardium was intimately adherent to the heart, and that there was a rounded opening on the inner surface of the right ventricle admitting the little finger, and a corresponding hole in the inter-ventricular septum leading into the left ventricle.

Turning, however, to the more common cases in which life was only preserved for shorter periods of time, we may refer to a case recorded in General Gordon's journals at Khartoum: "One man received a wound in the chest; he lived eleven days, and died. The doctor found a bullet lodged in the centre of the heart, in wall of ventricle. The doctor has this heart in spirits. It was a ball weighing the same as our Martini-Henry bullet."

Brigade-Surgeon Curran relates the case of a soldier struck at Corunna in 1809 to the left of the sternum, between second and third ribs. He was insensible for about an hour, was carried on board ship, and received no surgical attention beyond the application of a piece of plaster during the voyage. On arrival, the external wound was suppurating healthily. He was restless, had pain in the chest, his respiration was frequent and laboured, and his pulse was 120. He died the third day after landing, and fourteen days after the receipt of the wound. The right ventricle had a transverse opening about an inch in length, which penetrated its anterior surface near the origin of the pulmonary artery, and the tricuspid valve had a circular lacerated opening in it. The ball had evidently remained in the auricle all the time. Several other cases are recorded of people having lived for a time after bullet-wounds of the heart.

Coming to shorter survivals there is the case of the murder of the Duc de Berri in Paris in 1820. Here a dagger pierced the left ventricle, yet the duke lived eight hours. In many cases, no doubt, the direction of the wound at the part of the heart injured has much to do with the rapidity with which death occurs, but the size of the wound has necessarily more influence in this regard than mere direction.

It is, however, very important from a medico-legal point of view to remember that after even large wounds of the heart patients have been known to walk or run some considerable distance after the receipt of the injury. An instance is on record in which a stag, the auricle of whose heart had been practically destroyed, ran fifty or sixty yards. Taylor relates the following case: The keeper of a brothel was tried in Glasgow in the year 1819 for the murder of a sailor by shooting him through the chest. The auricles and part of the aorta next to the heart were "shattered to atoms" by the slugs and brass nails with which the piece was charged; and, in the opinion of the medical witnesses, the deceased must have dropped down dead on the moment that he received the shot. The body was found in the street, and the door of the prisoner's house was *eighteen feet* up an entry; so that it followed, if the medical opinion was correct, that the prisoner must have run after the deceased, and shot him in the street. It was, however, urged, and proved, that he had shot the deceased through the door of his own house, while the latter was attempting to enter by force. There was, in fact, a stream of blood from the door to the spot where the body lay. The prisoner was acquitted.

But many very extraordinary instances of the persistence of life after injury to the heart are on record; for example, one of a man who lived for twenty days with a skewer traversing the heart from side to side, and another of a boy who lived for five weeks with a piece of wood three inches long in his right ventricle.

